# The scar: the wind in the perfect storm—insights into the mysterious living tissue originating ventricular arrhythmias

**DOI:** 10.1007/s10840-021-01104-w

**Published:** 2022-01-24

**Authors:** C. Pandozi, Marco Valerio Mariani, C. Chimenti, V. Maestrini, D. Filomena, M. Magnocavallo, M. Straito, A. Piro, M. Russo, M. Galeazzi, S. Ficili, F. Colivicchi, P. Severino, M. Mancone, F. Fedele, C. Lavalle

**Affiliations:** 1 Department of Cardiology, San Filippo Neri Hospital, Rome, Italy; 2 Department of Cardiovascular, Respiratory, Nephrological, Aenesthesiological and Geriatric Sciences “Sapienza” University of Rome, Viale del Policlinico 155, 00161 Rome, Italy; 3 ASP, Ragusa Maggiore Hospital, Modica, Italy

**Keywords:** Scar, Ventricular arrhythmia, Catheter ablation, Cardiac magnetic resonance

## Abstract

**Background:**

Arrhythmic death is very common among patients with structural heart disease, and it is estimated that in European countries, 1 per 1000 inhabitants yearly dies for sudden cardiac death (SCD), mainly as a result of ventricular arrhythmias (VA). The scar is the result of cardiac remodelling process that occurs in several cardiomyopathies, both ischemic and non-ischemic, and is considered the perfect substrate for re-entrant and non-re-entrant arrhythmias.

**Methods:**

Our aim was to review published evidence on the histological and electrophysiological properties of myocardial scar and to review the central role of cardiac magnetic resonance (CMR) in assessing ventricular arrhythmias substrate and its potential implication in risk stratification of SCD.

**Results:**

Scarring process affects both structural and electrical myocardial properties and paves the background for enhanced arrhythmogenicity. Non-uniform anisotropic conduction, gap junctions remodelling, source to sink mismatch and refractoriness dispersion are some of the underlining mechanisms contributing to arrhythmic potential of the scar. All these mechanisms lead to the initiation and maintenance of VA. CMR has a crucial role in the evaluation of patients suffering from VA, as it is considered the gold standard imaging test for scar characterization. Mounting evidences support the use of CMR not only for the definition of gross scar features, as size, localization and transmurality, but also for the identification of possible conducting channels suitable of discrete ablation. Moreover, several studies call out the CMR-based scar characterization as a stratification tool useful in selecting patients at risk of SCD and amenable to implantable cardioverter-defibrillator (ICD) implantation.

**Conclusions:**

Scar represents the substrate of ventricular arrhythmias. CMR, defining scar presence and its features, may be a useful tool for guiding ablation procedures and for identifying patients at risk of SCD amenable to ICD therapy.

## Introduction

Myocardial scar provides the substrate for the majority of ventricular arrhythmias (VA). Although in electrophysiology dense scar is defined as an area with abnormal signals with an amplitude < 0.5 mV [1], low-voltage areas may be present in several anatomo-pathological settings, such as fibrosis, fat and oedema. Hence, the interpretation of the term scar may be misleading. Actually, the term scar does not identify a disease but the result of cardiac remodelling process that characterizes several cardiac pathologies. Indeed, cardiac scars are dynamic living structures where the extracellular matrix is full of phenotypically different groups of alive cells [2], making the scar a metabolically dynamic tissue with passive and active mechanical properties as well as an important electrophysiological activity due to the electrical and structural remodelling that takes place inside the living scar itself. In other words, scarring occurring in the setting of diverse heart diseases creates living, electrically and mechanically active tissues and not electrically insulated unexcitable structures [3]. This review discusses the anatomical features of scar, the role of imaging techniques in their detection and the importance of specific scar features in determining the electrophysiological derangement leading to the perfect storm that results in the initiation and maintenance of ventricular tachycardia (VT).

### Histopathology of scar

Pathology of scar includes different histologic components as interstitial and replacement fibrosis, surviving cardiomyocytes and other cell types (i.e. macrophages, lymphocytes, adipocytes), which are variously represented in ischemic and non-ischemic cardiomyopathies. On the basis of the distribution and homogeneity, four different patterns of fibrosis are usually described: compact, patchy, interstitial and diffuse.

In ischemic heart disease (IHD), necrotic cardiomyocytes are cleared by macrophages, and replacement fibrous tissue, consisting of collagen, is deposited by fibroblasts. Ischemic cell necrosis leads to extensive areas of replacement fibrosis, proceeding from the subendocardium to the subepicardium. A mixed process of myocyte resorption and collagen deposition results in islands of surviving myocardial cells surrounded by infarct scar [4].

Different amounts and locations of viable myocardium (sub-endocardial, epicardial or transmural), depending on the time to reperfusion and pre-existing collateral arteries, and various patterns of fibrosis result in complex geometries of scars [5]. In non-reperfused patients, ischemic insult produces a homogeneous dense scar surrounded by a small scar border zone [6], with uniform transmural necrosis as core infarct region [7]. Conversely, early reperfusion therapy during acute myocardial infarction (AMI) has been associated with less dense and less confluent electroanatomic scars. Although reperfusion therapy during AMI results in myocardial salvage and improves ventricular function, it clearly affects the arrhythmogenic substrate of VT by modifying scar architecture and VT circuit: the spatially heterogeneous healing process resulting in islands of surviving myocardial cells within healed infarct scars leads to the presence of a larger border zone interspersed around small areas of dense scar which is the substrate for channels of reentry circuits [8]. Hence, reperfusion therapy increases scar heterogeneity, number of VT channels and therefore scar arrhythmogenicity, influencing ablative approaches [9]. Due to the shorter VT cycle length, nowadays ischemic scar-related VTs are fast, unstable, haemodinamically not tolerated and unmappable by classic entrainment techniques in almost 90% of cases, requiring a substrate-based approach relying on voltage mapping and elimination of all abnormal and late potentials within the infarct zone [10].

Interestingly, post-infarction myocardial remodelling may result in the development of calcifications or lipomatous metaplasia inside the infarct zone or within scar borders, enhancing the propensity for VT after AMI. Baroldi et al. [11] found lipomatous metaplasia in almost 70% of patients with healed infarction, and Pouilopoulos et al. [12] demonstrated in infarct sheep model the role of intramyocardial adipose tissue in VT inducibility, altering electrophysiological properties such as conduction velocity. On the same line, Alyesh et al. [13] found myocardial calcifications in 70% of patients with post-AMI VT and postulated that calcifications may represent unexcitable tissue creating fixed barriers associated with re-entrant VT in post-infarction patients. Hence, beyond fibrosis, adipose tissue and calcifications may constitute the arrhythmogenic substrate of post-infarction VT [14].

The typical infarct scar is substantially different from non-ischemic cardiomyopathy scars, where the fibrotic areas are rarely compact; often, non-transmural and less likely sub-endocardial and cardiomyocytes are often more represented, even if morphologically altered (i.e. atrophic, hypertrophic, degenerated).

**Fig. 1:**
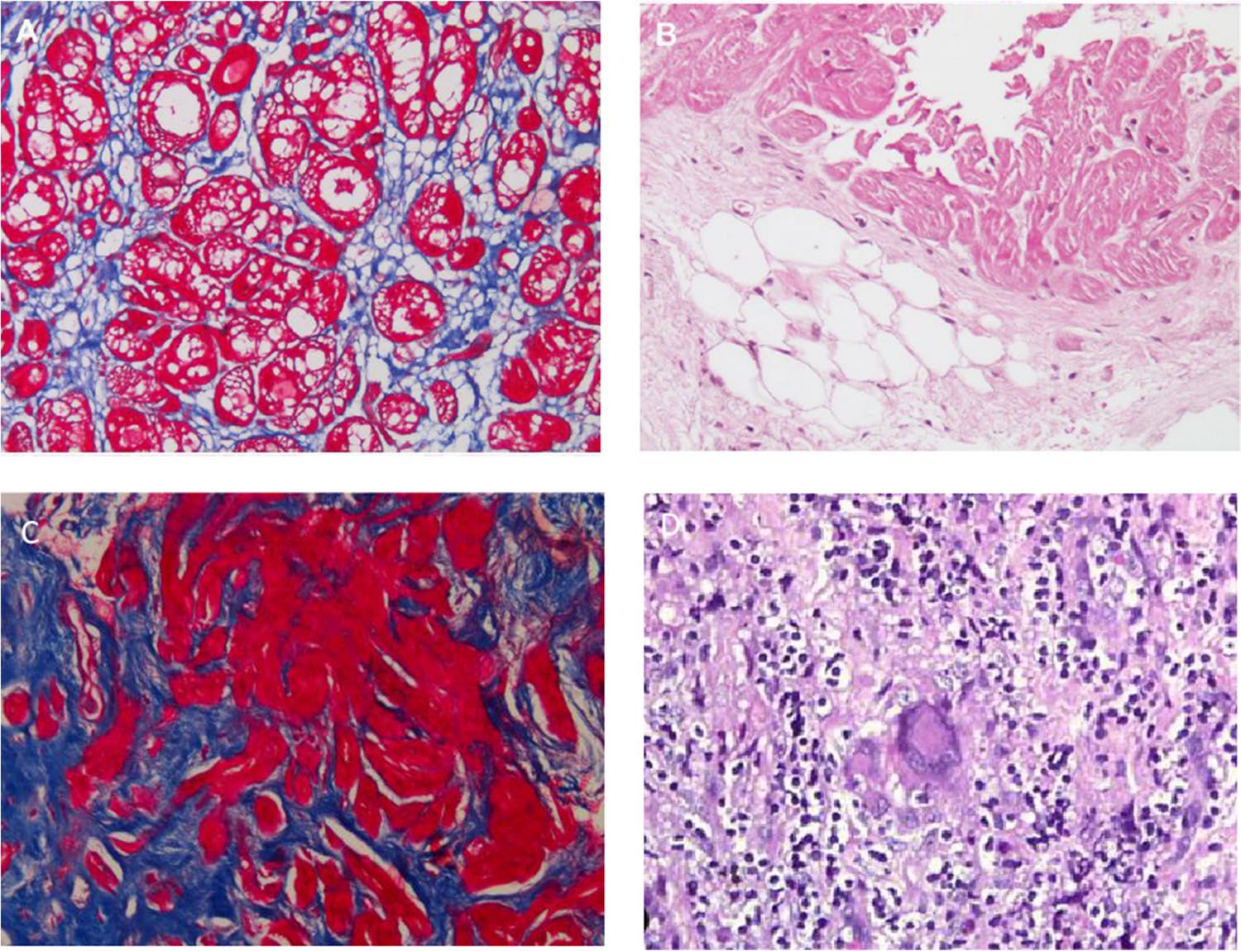
**A** Left ventricular endomyocardial biopsy from a patient with idiopathic dilated cardiomyopathy. Increased interstitial fibrosis (blue staining) and cardiomyocytes with severe reduction of myofibrillar content is evident (red-stained cells with vacuolization of cytoplasm). Masson trichrome staining, 200 × magnification. **B** Right ventricular endomyocardial biopsy from patient with ARVD. Cardiomyocytes are bordered by a huge area of fibrofatty replacement haematoxylin and eosin staining, 200 × magnification. **C** Left ventricular endomyocardial biopsy from a patient with hypertrophic cardiomyopathy. Replacement fibrosis (blue staining) interrupting cardiomyocytes in short runs. Masson trichrome staining, 200 × magnification. **D** Left ventricular endomyocardial biopsy from a patient with sarcoidosis. Inflammatory infiltration with the evidence of a multinucleated giant cell with arcuate arrangement of nuclei (Langhans type). Hematoxylin and eosin, 200 × magnification

In addition to focal areas of replacement fibrosis, cardiomyocyte degeneration with reduction of myofibrillar content and increased interstitial fibrosis is evident (Fig. 1A).

In myocarditis focal inflammatory infiltrates with necrosis of the adjacent cardiomyocytes are associated with various amounts of interstitial and replacement fibrosis. Myocarditis may lead to microaneurysms formation where the presence of inflammatory infiltrates with intense myocytolysis and large areas of fibrosis may be considered a pathogenetic substrate of ventricular arrhythmias [15].

The scar in arrhythmogenic cardiomyopathy (AC) is characterized by the presence of cardiomyocyte atrophy and fibrofatty replacement that involves not only the mid-myocardial layer but also the subepicardium with a highly specific distribution pattern that has been described as ringlike at CMR [16, 17]. The surviving myocardial fibres are embedded in fibrous tissue and fat and form a complex network of connections with the normal myocardium at the border of the dysplastic area (Fig. 1B).

In hypertrophic cardiomyopathy, areas of scar-like fibrosis are associated with hypertrophied cardiomyocyte in disarray interrupted in short runs by increased interstitial fibrous tissue and with microvasculopathy (Fig. 1C) [18].

VAs and SCD are often the principal manifestations of cardiac sarcoidosis, a multisystem, granulomatous disease caused by immunological response in genetically susceptible persons [19]. The biopsy shows noncaseating granulomas in the myocardium that represent anatomical barriers to depolarization propagation, hence possibly resulting in re-entrant VT (Fig. 1D).

Although Brugada syndrome (BrS) has been historically classified as an electrical disease, recent findings showed that electrical alterations may be caused by structural abnormalities mostly localized to the epicardial layer of right ventricular outflow tract (RVOT), driven by myocardial inflammation. Indeed, Pieroni et al. showed pathologic findings with myocardial inflammation in almost 80% of BrS patients who undergone endomyocardial biopsy [20].

As described above, the term “scar” includes various types of histopathologic patterns that could play different roles in the genesis of ventricular arrhythmias.

## Electrophysiology and arrhythmogenic potential of scar tissue

### The perfect storm for the initiation and maintenance of re-entrant (and non-re-entrant) ventricular arrhythmias

The presence of unidirectional block and slowing of conduction velocity are needed for the activation of the circuit and for re-entrant arrhythmias occurrence [21]. Furthermore, for the induction of sustained ventricular arrhythmias, the presence of a trigger [22] that stresses the electrophysiological substrate is necessary.

**Fig. 2:**
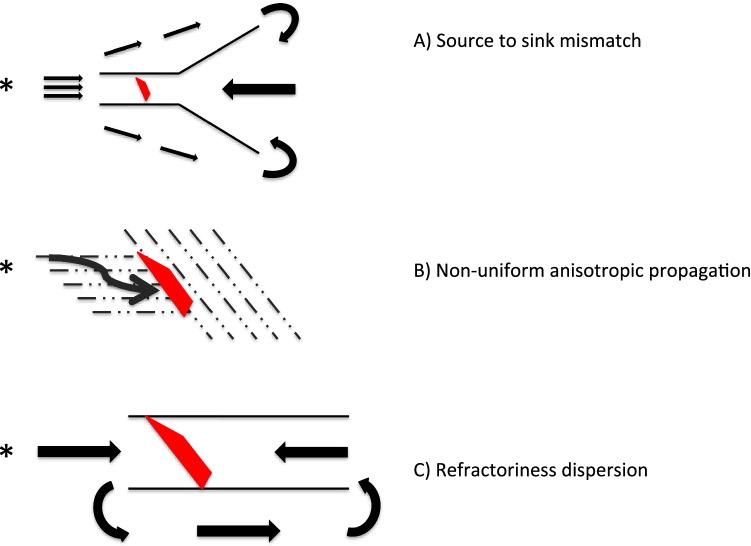
Electrophysiological mechanisms responsible of initiation and maintenance of re-entrant VT. **A** If the activation wavefront propagates from a source region with a relatively small volume and the tissue going forward, the sink is substantially larger in volume; the propagation will slow and even block because of the insufficiency of available current from the source to activate the larger-volume sink. Conduction is possible in the opposite direction because of the favourable sink to source relationship, allowing reentry to occur. **B** In non-uniform anisotropic propagation typical of scarred tissue, block can occur in regions of abrupt change in fibres orientation relative to the direction of propagation. **C** The wavefront generated by the ectopic beat finds the tissue with longer refractoriness still refractory and blocks. Hence, the wavefront is forced around the site of block and eventually invades the tissue retrogradely, when it has recovered its excitability. Arrows indicate direction of wavefront propagation; red dots indicate conduction of block sites

In a scarred tissue, there are several conditions that lead to unidirectional conduction block of a premature beat arising inside or outside the scar and to a slowing of conduction velocity. Unidirectional conduction block may arise because of reduced safety factor for conduction due to a source-sink mismatch [23, 24], peculiarities of non-uniform anisotropic conduction [25] or spatial disparity of refractory periods [26, 27] (Fig. 2).

A mechanism of conduction block of an ectopic beat is represented by the sink-source mismatch [23, 24]. An important electrophysiological feature of scarred tissue is represented by the different conduction properties between inhomogeneous tissue volumes such as those constituted by muscle fibres embedded within strands of fibrous tissue of different sizes [23, 24]. If, in the travel direction, the subsequent localized volume of conducting tissue, the sink, is smaller as compared with the source, propagation of the activation wavefront will proceed; there is sufficient electrical current available to conduct in the distal direction, and the wavefront actually accelerates. When the source and sink are of the same size, the wavefront propagates with no change in speed. However, if the activation wavefront propagates from a source region with a relatively small volume and if the tissue going forward, the sink, is substantially larger in volume, the wavefront will slow or even block because of the insufficiency of available current from the source to activate the larger-volume sink (source-sink mismatch) (Fig. 2A). It is important to underline that the source-sink balance is asymmetrical, that is, when propagation precedes in the opposite direction from the broadband to the narrow bundle, the source is larger than the sink and conduction succeeds [28].

In non-uniform anisotropic propagation typical of scarred tissue, block can occur in regions of abrupt change in fibre orientation relative to the direction of propagation. These branching sites represent areas where the safety factor for conduction is low as demonstrated by the failure of propagation at these sites of premature beats [29]. Normal cardiac tissue presents uniform anisotropy [29]; however, another kind of anisotropy exists and is called as non-uniform because, although a tight electrical coupling between cells in the longitudinal direction is present, side-to-side electrical coupling of adjacent groups of parallel fibres in the transverse direction is reduced or absent due to the interposition of strands of connective tissue inside or in the border zone of a compact scar transverse propagation may be interrupted [29, 30], and consequently adjacent bundles are excited in a “zigzag” sequence resulting in slow conduction with the consequent recording of fractionated extracellular electrograms (Fig. 2B) [30].

Finally, refractoriness dispersion inside the scar or difference in refractory periods between the scarred and healthy tissue is common in diseased hearts [26, 27]. Membrane current downregulation often occurs in diseased and scarred cardiac tissue leading to refractoriness dispersion, unidirection block and/or slow conduction (Fig. 2C). Surviving cardiomyocytes in the viable border zone adjacent to a prior infarction have signs of reduced excitability and sodium channel downregulation [31]: reduced action potential amplitude (phase 0 amplitude), reduced dV/dtmax (upstroke velocity) and slow recovery of excitability (postrepolarization refractoriness) [32]. Unidirectional block is also favoured by refractoriness heterogeneity due to spatially heterogeneous K channel downregulation coupled with post-repolarization refractoriness. Besides active membrane ionic currents downregulation and specifically alterations in the fast inward sodium current, cellular uncoupling caused by cardiac fibrosis leads to refractoriness dispersion in other ways. First, fibrosis cancels the homogenizing effects on repolarization that occurs in well-coupled cells, where the current flow during repolarization will tend to decrease dispersion by prolonging action potential with a short duration and shortening action potential with a long duration [33]. In scarred tissue, the high coupling resistance reduces the electrotonic interaction flow among myocytes resulting in increased repolarization heterogeneity [33]. Second, in fibrotic areas, nerve fibres may be interrupted, causing regional supersensitivity to circulating catecholamines and acetylcholine and thus creating inhomogeneity in refractoriness [27].

For what concerns conduction velocity, some of the factors illustrated above leading to conduction block, when present in a less marked way, are often responsible of the reduced conduction velocity recorded in the scarred tissue. Both active membrane properties and passive axial resistivity involved in cardiac conduction velocity and propagation are affected by electrophysiological and anatomic remodelling induced by the heart diseases [29]. In fact, sodium channel remodelling, connexin expression remodelling, non-uniform anisotropic conduction related to the development of fibrosis and an impedance mismatch of a mild degree that does not lead to conduction block affect conduction velocity slowing propagation in the scarred tissue [23, 29, 32]. In particular, in scarred tissue of hypertrophied, ischemic or failing heart, conduction velocities are further depressed by downregulation of gap junction proteins (connexins) [34, 35]. Downregulation of Cx43 causes loss of intercalated disks and/or their redistribution along the long axis of the fibres (lateralization). This lateralization increases axial resistivity and further reduces longitudinal conduction velocities without improving transverse conduction velocities [36]. In conclusion, unidirectional conduction block and low conduction velocity are hallmarks of fibrous tissue; they may originate by similar electrophysiological and anatomical derangements of different degrees and lead to the likely activation of anatomical or functional re-entrant circuits.

For the induction of a sustained ventricular arrhythmias, the presence of a trigger [22] that stresses the electrophysiological substrate is necessary. The trigger of ventricular arrhythmias is represented by ectopic beats caused by abnormal automaticity and triggered activity due to early and delayed afterdepolarizations. Fibroblasts play a prominent role in the development of triggers of ectopic beats inside the scar. Resident cardiac fibroblasts are in normal conditions non-excitable cells of mesenchymal origin that produce interstitial collagen, with little or no contractile microfilaments [37]. During scar formation, fibroblasts undergo phenotype transition into myofibroblast, showing migratory capability and connexin expression, and can form, at least *in vitro*, homocellular gap junction with each other as well as heterocellular gap junction with myocytes [38, 39]. Heterocellular gap junctions influence myocyte electrophysiology due to the depolarizing effect of myofibroblast on the negative myocyte resting membrane potential that become less negative and then prone to spontaneous diastolic depolarization and automaticity [40]. In scarred tissue, also the source-sink effect (impedance mismatch) has an important role in promoting triggers. In fact, in normal myocardium, only when a large number of neighbouring myocytes synchronously depolarize, a sufficient current is generated to depolarize the nearby myocytes allowing impulse propagation and the origin of a premature ectopic beat that depolarize the whole heart. However, in scarred tissue, the number of myocytes required to trigger an ectopic beat decreases dramatically because the presence of fibrous tissue changes the 3-dimensional arrangement of normal myocardial tissue into a 2 or even 1-dimensional structure where the current sink has to propagate to an area or a line of neighbouring myocytes instead of 3-dimensional volume as in normal myocardial tissue and where the available current from the source is sufficient to activate the limited number of cells that form the smaller area or line of the sink [41]. In this situation, only a very limited number of misbehaving myocytes are capable to trigger a premature ventricular beat because of a positive source-sink relationship (Fig. 2A).

Re-entrant circuits and VTs channels may be either anatomically defined, fixed and present in sinus rhythm or functional, present during VT but not in sinus rhythm. However, several studies, both in animals and in humans VTs, have demonstrated the presence of functional circuits unassociated with anatomical defined barriers. These papers have shown that initiation of sustained monomorphic VT requires the development of unidirectional block and the formation of lines of functional block creating the borders for the diastolic pathway in areas of slow conduction [42]. Several mechanisms have been proposed to explain the development of functional block including the defect in the number and function of connexin consisting gap junctions [43], regional differences in ionic currents in cells of the border zone [44], leading to alteration of the depolarization and repolarization phases of the action potential and then to the inhomogeneous changes in refractory periods and refractoriness dispersion. In other words, while in the anatomically defined reentry, the circuit and the isthmus are defined by anatomic barriers already present in sinus rhythm (strand of connective unexcitable tissue), and its activation is related to unidirectional block and slow conduction, in functional reentry even the definition of the circuit, that is the isthmus and the lateral barriers, is determined by conduction block occurring only during conduction stress conditions (premature beat) and not present during sinus rhythm. In functional reentry, active membrane properties and passive axial resistivity changes inside the scar determine both the development and the activation of the circuit [45].

### Electroanatomical mapping

Among mapping techniques used to identify discrete site of ablation for VT, bipolar substrate mapping based on electrogram peak-to-peak voltage analysis during sinus rhythm distinguishes normal myocardial tissue (voltage > 1.5 mV) from the border zone (0.5 mV < voltage < 1.5 mV) and dense scar (voltage < 0.5 mV), while unipolar substrate mapping provides tissue characterization of epicardial and mid-myocardial layers [1], with normal values > 8.3 mV and abnormal values < 5.5 mV. However, abnormal substrate is identified not only by low-voltage areas but also from late potentials (LPs) and local abnormal ventricular activities (LAVAs). Of note, LPs were defined by Cassidy et al. [46] as any abnormal, fractionated electrogram that persists beyond the duration of surface QRS, whereas Jais et al. [47] described LAVA as sharp, high-frequency near-field signals of slowly conducting tissue, potentially VT isthmuses. Therefore, current criteria to define abnormal substrate are based on the association of abnormal electrograms (wide, late and fractionated) with low tissue voltage. In the last years, the evolution of electroanatomical mapping systems and catheter technologies, such as ripple mapping and ultra-high-density mapping, made possible a detailed assessment of VT re-entrant circuit, even to the definition of discrete conducting channels inside the scar. Luther et al. described the use of ripple mapping to identify the conducting channels in post-infarct ventricular scars [48]. Ripple mapping is a 3-dimensional activation visualization that shows each electrogram component as a dynamic bar that protrudes from its site on the surface geometry. The movement of the bars represents the direction of propagation and constitutes the activation map. This approach to definition of VT ablation targets simultaneously provides structural and functional data on VT substrate, because ripple activation can be superimposed on a bipolar voltage map, showing the surface geometry with both voltage and activation at the same time. More recently, Martin et al. [49] reported a series of ultra-high-density mapping-guided VT ablations using the diagnostic multipolar catheter Orion (Intellamap Orion, Boston Scientific). The use of ultra-high-density mapping catheter was associated with an excellent definition of VT circuit conduction velocity, voltage and topography, identifying VT isthmuses, entrances, exits and dead ends. Nowadays, all these technological means make electroanatomical mapping of uppermost importance in the management of scar-related VT.

However, although substrate-based mapping and ablation approaches resulted effective in achieving VT control, electrogram abnormalities do not mean fibrosis because substrate mapping cannot provide tissue characterization. This concept has been clearly outlined by Samanta et al. [50] that showed that low-voltage areas detected when mapping within infarct may not accurately represent fibrosis or scar but fat, thus suggesting the importance of preprocedural imaging techniques in providing additional characterization of the arrhythmogenic substrate before ablation procedure. Nevertheless, electroanatomical mapping still represents the gold standard for VT-related substrate definition [51] and localization. Electrocardiographic imaging (ECGI), using up to 252 electrodes at torso level and heart reconstruction by thoracic computed tomography (CT), has been used to guide treatment of ventricular ectopy and localization of site of origin of VT for stereotactic radioablation [52]. Graham et al. [53] showed that ECGI yielded higher accuracy in providing a region of interest for targeting mapping and ablation as compared to 12-lead ECG-based algorithm; however, it lacks adequate accuracy and precision to guide catheter ablation without ancillary detailed electroanatomical mapping.

### Imaging of scar

**Fig. 3:**
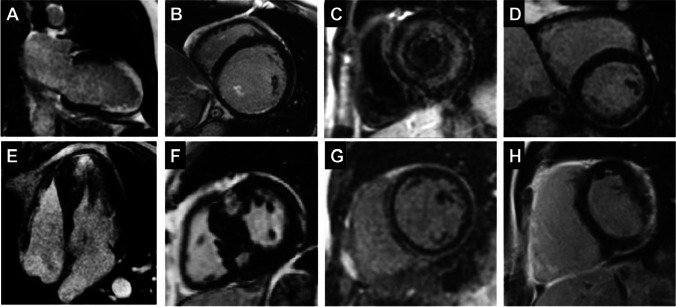
Examples of LGE pattern. **A** Ischemic LGE pattern, mainly transmural, involving apex and apical cap, anterior and inferior wall at the mid segment; **B** sub-endocardial LGE (< 50%) at the lateral wall, note the LGE involving the postero-medial papillary muscle; **C** diffuse sub-endocardial LGE in patient with cardiac amyloid; **D** sub-epicardial LGE involving lateral and inferior wall in a patient with myocarditis; **E** non-ischemic LGE in the area of maximal hypertrophy in a patient with apical hypertrophic cardiomyopathy (HCM); **F** patchy areas of LGE at the septum at the right ventricle insertion points in patients with HCM; **G** non-ischemic LGE involving the inferior wall (sub-epicardial) and the septum (midwall) in a patient with dilated cardiomyopathy; **H** non-ischemic LGE involving the infero-lateral wall and the RV free wall in a young patient with arrhythmogenic cardiomyopathy with involvement of both ventricles

Cardiovascular magnetic resonance (CMR) is considered the gold standard imaging test for non-invasive tissue characterization. Late gadolinium enhancement (LGE) allows the detection of myocardial fibrosis, and, although not disease specific, its presence, distribution and extent widely differ and allow the distinction among ischemic and non-ischemic diseases [54] (Fig. 3). Indeed, location, pattern and transmural of LGE may be diagnostic of specific diseases. After an ischemic event, LGE can be sub-endocardial or transmural (following the “wavefront phenomenon” of necrosis from subendocardium to subepicardium). Conversely, a wider spectrum of patterns can be observed in the non-ischemic cardiomyopathy including mid-wall, sub-epicardial, diffuse or patchy LGE [55]. In particular, mid-wall LGE is a typical finding in non-ischemic cardiomyopathy and heralds a poor prognosis in terms of survival and propensity to VT and SCD because it represents an advanced stage of myocardial derangement and cardiac detrimental remodelling [56].

However, LGE and fibrosis are not synonymous because LGE is not only a marker of replacement fibrosis, but it can be related to a wider spectrum of myocardial tissue changes including cardiac amyloid, fat infiltration and oedema [54]. In the last years, CMR has become crucial in the evaluation of patients presenting with VA, being able to define site, localization and transmurality of the arrhythmic substrate and therefore able to guide catheter ablation procedures. Moreover, the association between scar size and features and VT development, applying CMR and electroanatomical voltage maps, has been shown [57]. In IHD, Bello et al. [58] demonstrated that patients with larger infarct mass had higher rates of VT inducibility, while Schmidt et al. [59] found that border zone mass, detected by CMR, was the best predictor of VT inducibility in patients undergoing ICD implant. Indeed, border zones represent areas of tissue heterogeneity characterized by islands of surviving myocardial cells within fibrosis and are the anatomic substrate for VT. Roes et al. determined infarct grey zone using the maximum signal intensity (SI) within the infarct region [60]. The infarct core was defined as myocardium with ≥ 50% of maximum SI, whereas the grey zone was defined as myocardial region with SI ≥ 35% but with SI < 50%. The authors demonstrated that infarct grey zone, as surrogate of infarct tissue heterogeneity, was the strongest predictor of spontaneous VA among other clinical and CMR variables (total infarct size and ventricular function and dimension) in patients with previous myocardial infarction. Therefore, the presence and quantification of these grey zones within myocardial tissue have prognostic implications.

Finally, small studies have shown the possible role of CMR in predicting the discrete site of ablation identifying conducting channels relevant to VT development and maintenance [61–63]. Andreu et al. [61] processed LGE-CMR sequences by a dedicated software (ADAS-VT, Galgo Medical, Barcelona, Spain), which, applying a pixel signal intensity-based algorithm to characterize hyperenhanced areas as scar, provided a pixel signal intensity map projected to ventricle shells. The shells were then imported into the navigation system and merged with electroanatomical map to guide ablation. This tool demonstrated to be promising in the study by Andreu et al., as CMR-aided scar dechannelling was associated with lower VT-recurrence rates after radiofrequency ablation.

In the study by Casella et al. [64], CMR and electroanatomic mapping showed similar accuracy, with high degree of correlation among the techniques. CMR proved similar sensitivity as compared to electroanatomic voltage mapping (77% vs 74%) in identifying myocardial pathological substrate in 162 patients undergone endomyocardial biopsy; tough CMR yielded lower specificity (47% vs 70%). At the end of the day, unravelling cardiac scar is the future direction of CMR, providing the distinction between VT and non-VT supporting channels in order to target only discrete arrhythmogenic zones with limited ablative approaches, leading to more effective and safe procedures.

The majority of patients referred for VT ablation and CMR have an implantable cardioverter defibrillator (ICD) which is associated with artefacts that undermine image integrity and CMR utility. The artefact size depends on generator position, dimension and on the distance between generator and the heart [65]. LGE-CMR sequences are the most affected by artefacts that are more often located to the anterior and apical myocardial regions. Stevens et al. [66] proposed the use of wideband LGE sequence to reduce the amount of hyperintensity artefact due to a frequency shift when applying the standard inversion pulse. This technique strongly reduced artefact size and improved LGE-CMR image quality, resulting in significant correlation among scar size measurements using CMR and electroanatomical mapping. When CMR is unsuitable or unavailable, multidetector cardiac computed tomography (MDCT) is a valuable alternative providing definition of the arrhythmogenic substrate by means of different image characteristics as wall thinning, decreased perfusion, hyperattenuation and hypoattenuation, thus representing an adjunctive tool for VT ablation. Recently Takigawa et al. have shown the relationship among wall thickness channels derived from MDCT and VT isthmuses as defined by high-density activation mapping: electrical VT isthmuses co-localized with 1 or more of the MDCT channels in 100%, and 50% of MDCT channels corresponded to VT isthmuses [67]. However, these results need to be confirmed in larger population.

## Clinical implications

Yearly, in European countries, 1 per 1000 inhabitants dies for sudden cardiac death (SCD) mainly as a result of ventricular arrhythmias [68]. ICD therapy confers a significant survival benefit and should be reserved to patients at high risk of arrhythmic events after accurate risk stratification.

Although currently left ventricular ejection fraction (LVEF) plays a central role in selecting patients for ICD therapy in primary prevention, several evidences demonstrated that LVEF criterion is not the right determinant of the utilization of ICD for the prevention of SCD. First, both in patients with non-ischemic and ischemic cardiomyopathy, LVEF has shown low specificity for the prediction of SCD. Hence, reduced LVEF identifies patients at increased risk not only for SCD but also for non-sudden death, and this is not surprising because LVEF has not direct relation to any mechanism leading to VT/VF [69]. Second, among patients deemed to be at high risk of SCD on the basis of LVEF, only a small portion receives appropriate therapies from the device during follow-up, whereas a lot of patients will not benefit from the ICD or will experience device-related complications. Third, several trials showed that high-risk patients with low LVEF have an overall low risk of SCD, and the implant of ICD would not be cost-effective. Lastly, SCD often occurs in patients with preserved or only moderately reduced LVEF. This group of patients, despite the lower relative risk, accounts for the greatest number of SCD events larger than the group of patients with severely depressed systolic function. Using LVEF as risk stratifier for SCD implies missing the majority of SCD cases with the result of under-treating patients at risk of arrhythmic death.

In consideration of the pivotal role of the scar in the genesis of ventricular arrhythmias, several studies have investigated the characterization of the scar through imaging modalities, especially CMR by LGE technique, as a tool for the targeted stratification of SCD risk. Boyè et al. showed a strong association among the infarct transmurality by LGE and life-threatening arrhythmias in 52 patients with IHD meeting the inclusion criteria of MADIT trial [70]. In the same direction, Scott and colleagues found a significant association between LGE extent and appropriate ICD therapy during follow-up [71]. In 137 patients evaluated for ICD therapy, Klem et al. demonstrated that the assessment of myocardial scarring with LGE improved risk stratification for SCD regardless of CMR-LVEF [72]. Indeed, at multivariable analysis, scar size was an independent predictor of the composite primary outcome of death and appropriate ICD discharge, with a marked step up in event rate for scar size > 5% of LV mass. Infarct patients with LVEF > 30% and significant scarring had risk similar to patients with LVEF ≤ 30%, while among patients considered at high-risk for SCD on the basis of reduced LVEF (≤ 30%), the presence of myocardial scarring less than 5% of LV mass indicated a risk similar to that of patients with LVEF > 30%. The added risk stratification value of CMR was confirmed by Pontone et al. [73]. Among 409 patients with IHD and non-ischemic cardiomyopathy evaluated for primary prevention ICD therapy, patients experiencing major adverse cardiac events (defined as a composite endpoint of long runs of nonsustained ventricular tachycardia, sustained ventricular tachycardia, aborted SCD or SCD) had lower LVEF by echocardiography and CMR and higher LGE per-patient prevalence in comparison with patients without events. The use of CMR-LVEF and the presence of LGE provided a more accurate risk stratification in a subgroup of patients in which several missed-ICD implantations fall on the basis of TTE-LVEF criterion.

In a meta-analysis of 19 studies, Disertori et al. [74] demonstrated that LGE is a powerful predictor of arrhythmic risk in patients with ventricular dysfunction, irrespective of ischemic or non-ischemic aetiology. Furthermore, Di Marco et al. presented a systematic review and meta-analysis that demonstrated the strong and independent association between LGE and the risk of ventricular arrhythmia and SCD in 2948 patients (reported in 29 studies) with non-ischemic cardiomyopathy followed for an average of 3 years [75]. LGE was present in 44% of patients, and despite its association with arrhythmic endpoints was significant when considering studies including patients with LVEF > 35%, it was maximal in studies including patients with LVEF < 35%. In consideration of the low annual event rate in patients without LGE compared to those with LGE (2.1% vs 17.2% per year) and the similar proportion of arrhythmic events in patients with LVEF above and below 35%, LGE seems to be a powerful risk stratification tool, beyond LVEF.

Besides LGE, other CMR-derived tools may be useful for arrhythmic risk stratification. In particular, Puntmann et al. [76] showed that non-invasive measures of diffuse myocardial disease by T1 mapping sequence (native T1 and extracellular volume), as well as the presence and extent of LGE, were significant predictors of all-cause mortality in 637 consecutive patients with non-ischemic cardiomyopathy. However, at multivariable analysis, native T1 was the sole independent predictor of outcomes, underlining the crucial role of diffuse myocardial fibrosis as driver of arrhythmic outcome, acting as a potential VT substrate, in patients with early non-ischemic cardiomyopathy. This result suggests that T1 mapping, alongside LGE, may be a useful tool for arrhythmic stratification risk in patients with non-ischemic cardiomyopathy. Recently, Goult et al. [77] evaluated a new CMR technique, the CMR quantitative texture analysis, as tool for calculating mean entropy as marker of scar heterogeneity. This technique analyses the filtered LGE image and quantifies scar heterogeneity from the distribution of pixel intensities within a region of ventricular scar. The more the scar image becomes complex with several different pixel values detected, the more the entropy value increases. Goult et al. [77] showed that scar heterogeneity, quantified by mean entropy, was an independent predictor of appropriate ICD therapy in patients with ischemic cardiomyopathy and mixed cardiomyopathy, whereas in patients with non-ischemic cardiomyopathy, native T1 values were the sole predictor of arrhythmic outcomes.

It is desirable that in the future, the arrhythmic risk stratification should not be based on LVEF or scar extension only but based on methods able to evaluate the arrhythmogenicity of the scar. Meanwhile, new risk stratification tools will be developed and validated; a combined approach incorporating clinical variables and scar presence and features detected with LGE at CMR probably represents the best methodological approach to use [78].

## Conclusions

Scar is the fascinating substrate for the development of VA. Although it is usually considered a dead tissue, scar has dynamic electrophysiological properties addressing for its complexity and its several clinical implications. CMR represents a useful tool for identifying arrhythmic substrate and for programming catheter ablation. Several evidences underscore the suboptimal results achieved in the field of SCD prevention with current adopted strategies, mainly based on LVEF. In consideration of the pivotal role of scar in the development and maintenance of VA, future studies should address the prognostic power of CMR-based scar characterization in selecting patients at risk of SCD and amenable to ICD implantation.
